# Analysis of COVID-19 pandemics in Kazakhstan

**DOI:** 10.34172/jrhs.2021.52

**Published:** 2021-05-26

**Authors:** Baurzhan Zhussupov, Timur Saliev, Gulya Sarybayeva, Kuanysh Altynbekov, Shynar Tanabayeva, Sagat Altynbekov, Gulnara Tuleshova, Dainius Pavalkis, Ildar Fakhradiyev

**Affiliations:** ^1^National Center for Public Health, Nur-Sultan, Kazakhstan; ^2^S. D. Asfendiyarov Kazakh National Medical University, Almaty, Kazakhstan; ^3^Astana Medical University, Nur-sultan, Kazakhstan; ^4^Republican Scientific and Practical Centre of Mental Health of the Ministry of Health of the Republic of Kazakhstan, Almaty, Kazakhstan

**Keywords:** Comorbidity, COVID-19, Coronavirus, Epidemiology, Mortality

## Abstract

**Background:** This study aimed to analyze the demographic and epidemiological features of identified COVID-19 cases in Kazakhstan.

**Study design:** A cross-sectional study.

**Methods:** This cross-sectional study aimed to analyze COVID-19 cases (n=5116) collected from March 13 to June 6, 2020, in Kazakhstan. The data were obtained from a state official medical electronic database. The study investigated the geographic and demographic data of patients as well as the association of COVID-19 cases with gender and age. The prevalence of symptoms, the presence of comorbidities, complications, and COVID-19 mortality were determined for all patients.

**Results:** The mean ±SD age of the patients in this study was 34.8 ±17.6 years, and the majority (55.7%) of COVID-19 cases were male and residents of cities (79.6%). In total, 80% of the cases had the asymptomatic/mild form of the disease. Cough (20.8 %) and sore throat (17.1%) were the most common symptoms among patients, and pneumonia was diagnosed in 1 out of 5 cases. Acute respiratory distress syndrome (ARDS) was recorded in 1.2% of the patients. The fatality rate was 1% in the study population and lethality was 2.6 times higher in males compared to females. Each additional year in age increased the probability of COVID-19 infection by 1.06 times. The presence of cardiovascular, diabetes, respiratory, and kidney diseases affected the rate of mortality (*P*<0.05).

**Conclusions:** The results demonstrated a high proportion (40%) of the asymptomatic type of coronavirus infection in the Kazakhstan population. The severity of COVID-19 symptoms and lethality were directly related to the age of patients and the presence of comorbidities.

## Introduction

At the end of 2019, an unknown infectious disease outbreak was registered in Wuhan city of China ^1-3^ that has been caused by a new coronavirus named SARS-CoV-2. In February 2019, the World Health Organization (WHO) proposed the new name ‘COVID-19’ for the disease caused by this virus^4^. More than 53 million confirmed cases and more than 1.3 million deaths from COVID-19 have been registered worldwide since the beginning of the pandemic ^5-9^.

COVID-19 disease spread quickly throughout the countries and continents. It has been shown that COVID-19 is most contagious in the first 5 days after symptoms onset, indicating the critical role of early identification and patient’ isolation^10-13^. Moreover, timely screening for coronavirus infection allows the identification of infected patients with severe symptoms, people in risk groups, and possible asymptomatic virus carriers. Therefore, it can help to prevent the further spread of this highly contagious infection ^14^.

To date, there are several publications and studies related to coronavirus infection and its epidemiology ^15-19^. Since the beginning of the pandemic, many studies have identified risk factors, such as male gender, old age, and the presence of various comorbidities associated with high probability of morbidity and mortality from COVID-19 ^20-22^. However, data on the incidence and clinical course of COVID-19 in the Republic of Kazakhstan have not been fully collected and analysed ^23^. Up to date, there are some reports on COVID-19 infection in paediatric population of Kazakhstan ^24,25^ and the general public ^26-29^. The study of all features of disease manifestation and outcomes in the Kazakhstan population is of great theoretical and practical interest since COVID-19 is a new disease.

 This study aimed to determine the demographic and epidemiological characteristics of the detected cases of COVID-19 in the Kazakhstan population. In addition, the association of these factors with the clinical symptoms and adverse outcomes was assessed as well. The results of this study have a practical value due to the active detection of COVID-19 cases and high coverage of patients in the risk groups and sites of the infection.

 The transparent and complete report provided in this study may be valuable from a statistical and epidemiological point of view.

## Methods

###  Ethical issues

 Ethical approval was waived by the Institutional Review Board (S.D. Asfendiyarov Kazakh National Medical University, Almaty, Kazakhstan) due to the retrospective nature of the study. Data records were anonymous, and informed consent was not required.

###  Data collection

 In total, 5,223 cases of COVID-19 cases were analyzed during the study period (from March 13 to June 6, 2020). This study covers the data from 5,116 laboratory-confirmed COVID-19 cases collected from all 17 regions of Kazakhstan. The data were obtained from an official medical electronic database in the Ministry of Healthcare of the Republic of Kazakhstan. The information was extracted from electronic medical records (blinded) using a standardized data collection form. Missing dates and information were clarified and confirmed by direct communication with healthcare providers.

 The study included data from patients with a confirmed diagnosis based on a positive test. The determination of SARS-CoV-2 virus RNA (in a swab from the nose or throat) was conducted using real-time fluorescent polymerase chain reaction with reverse transcription (RT-PCR). The samples were taken from the patients with symptoms similar to those of COVID-19 who had a suspected exposure to SARS-CoV-2, as well as those with respiratory disease, and asymptomatic patients. Unconfirmed cases of COVID-19 with negative RT-PCR test results were excluded from the analysis.

 The geographical data and demographic characteristics of the studied patients (age, gender) were analyzed. The patients were divided into eight age groups of <10, 10-19, 20-29, 30-39, 40-49, 50-59, 60-69, and 70˂ years. In addition, the relationship between gender and age in the reported cases of COVID-19 was studied as well. The detection rate of COVID-19 cases was also determined in terms of the place of residence (city /village).

 The severity of the forms of COVID-19 disease was determined through the examination of clinical symptoms and radiological findings (e.g. Chest X-Ray and computed tomography (CT)).

 Based on the clinical data, the presence of respiratory and non-respiratory symptoms was studied in infected patients, and the comorbidities and the severity level of COVID-19 were also identified. The patients were divided into five groups of asymptomatic, mild, moderate, severe, and critical, in terms of the severity of the disease.

 The degree of involvement of lungs in the pathological process was determined using data from functional research methods (i.e., chest x-ray, computed tomography). Two independent radiological specialists examined and interpreted chest X-rays and CT scans. Moreover, the data on the incidence of complications such as the development of pneumonia and ARDS, the need for mechanical ventilation, as well as the relationship between case fatality rate and risk factors for COVID-19 were examined as well.

###  Statistical analysis

Data were analyzed statistically using R software (version 4.0.5) ^30^. Quantitative and qualitative (categorical) variables were presented by the mean±SD and absolute value and proportion. Contingency tables and logistic regression were used to produce odds ratios and adjusted odds ratios and analyze the relationships. The Gamma coefficients and the phi correlation coefficient were calculated to measure the strength of the association between the presence of symptoms and age group as well as the relationship between dichotomous variables (i.e. the presence of symptoms and gender).

The method of multiple imputations of missing data was used in order not to exclude cases with partially filled data. For the analysis, it was assumed that the fact of missing data is not related to their values ​​ (missing at random - MAR). Five imputed datasets were produced. Logistic regression coefficients and their respective standard errors were calculated from the pooled model using Rubin’s Rules ^31^.

## Results

The demographic and clinical characteristics of patients with COVID-19 are presented in Table 1. Males included the majority (55.7%) of the COVID-19 case. The mean age of participants was estimated at 34.8±17.6 years (31.8 years for males and 38.7 years for females). Moreover, the 20 -29 age group accounted for almost 30 % of all COVID-19 cases. The largest number of cases was registered in the cities of Almaty (36%) and Nur-Sultan (8.4%), Kazakhstan.

 In general, urban dwellers (79.6%) prevailed in all groups. The presence of respiratory symptoms at the time of registration was noted in only 36.3% of cases, while more than 60% presented with no symptoms at the time of diagnosis. At the same time, the asymptomatic form of COVID-19 was established in 40.9% of cases, which indicated the appearance of respiratory symptoms in more than 20% of cases after the diagnosis of COVID-19.

 The most common symptom of the COVID-19 disease (before diagnosis) included cough (20.8%), sore throat (17.1%), fever (11.6%), and running nose (7.2%). Other symptoms, such as shortness of breath, muscle pain, and diarrhoea were reported in less than 5% of cases. Asymptomatic, mild, moderate, severe and critical forms of disease were reported in 40.9%, 36.7%, 20.1%, 2.1% and 0.1% of the cases, respectively. Moreover, viral pneumonia was detected in 18.9% of the patients. Concomitant diseases were observed in 18.8% of cases, including hypertension (7.4%), respiratory diseases (4.6%), and anaemia (4%). Eventually, 49 (1.0%) cases of the disease were fatal.

**Figure 1:**
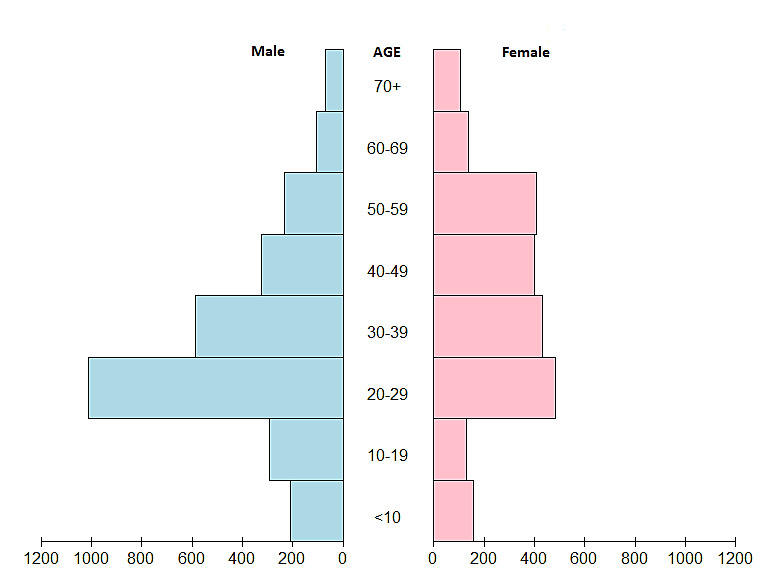
Pyramid of distribution of COVID-19 cases among genders and age groups

The age and gender pyramid reflects a high proportion of the 20-29 age group (Figure 1). Although the number of males exceeded the number of females in the younger age groups (up to 10 years old, 10-19, 20-29, and 30-39 years old), especially among 20 years olds, females were more prevalent in the older age groups.

**Table 1:** Demographic and clinical characteristics of COVID-19 cases

**Characteristics**	**Number**	**Percent(%)**
Gender		
Male	2849	55.7
Female	2267	44.3
Age (year)		
<10	369	7.2
10-19	419	8.2
20-29	1496	29.2
30-39	1016	19.9
40-49	725	14.2
50-59	644	12.6
60-69	242	4.7
Over 70	178	3.5
Region (population in the study, total %)		
Akmola region (736.2, 3.9%)	129	2.5
Aktobe region (883.6, 4.7%)	288	5.6
Alma-Ata's region (2059.1, 11.0%)	242	4.7
Atyrau region (647.4, 3.5%)	53	1.0
Eastern Kazakhstan region (1368.2, 7.3%)	62	1.2
Zhambyl region (1131, 6.1%)	219	4.3
Western Kazakhstan region (657.6, 3.5%)	340	6.6
Karaganda region (1376.6, 7.4%)	333	6.5
Kostanay region (867.9, 4.6%)	84	1.6
Kyzylorda Region (805.1, 4.3%)	189	3.7
Mangistau region (703, 3.8%)	127	2.5
Pavlodar region (752, 4.0%)	152	3.0
North-Kazakhstan region (547.7, 2.9%)	35	0.7
Turkestan region (2021.8, 10.8%)	311	6.1
Nur-Sultan city (1144.8, 6.1%)	428	8.4
Almaty city (1927.7, 10.3%)	1844	36
Shymkent (1042.2, 5.6%)	280	5.5
Residence		
Urban	4044	79.6
Rural	1037	20.4
Symptoms during registration of the disease
Fever	589	11.6
Sore throat	873	17.1
Rhinorrhoea	369	7.2
Cough	1063	20.8
Muscle pain	161	3.2
Diarrhoea	110	2.2
Dyspnoea	204	4.0
Disease severity		
Asymptomatic	2074	40.9
Mild	1863	36.7
Moderate	1018	20.1
Severe	109	2.1
Critical	6	0.1
Pregnancy	78	3.4
Co-morbidities		
Cardiovascular disease	285	2.6
Hypertension	380	7.4
Immuno-deficiency	7	0.1
Diabetes	128	2.5
Obesity	84	1.7
Kidney disease	100	2.0
Liver disease	61	1.2
Respiratory disease	230	4.6
Neurological disease	63	1.2
Malignancy	20	0.4
Anemia	205	4.0
Allergy	133	2.6
Complications		
Pneumonia	932	18.9
ARDS	61	1.2
Mechanical ventilation	64	1.3
Outcome		
Died	49	1.0

In total, 40% of all cases had COVID-19 symptoms at the time of registration of this disease. Moreover, the prevalence of symptoms increased with age (Table 2). In addition, the symptoms were observed only in 23.3 % of cases among patients under 10 years of age, while it was 53.4% in the 70 year-old-patients and above. The highest value of the Gamma coefficient (strength of the association between clinical symptom and age) was obtained for the shortness of breath (0.53), muscle pain (0.30), cough (0.24), and fever (0.23). Symptoms such as runny nose, sore throat, and diarrhea were significantly less age-related. In general, all symptoms were more common in females than males. This difference can be explained by the difference in age between males and females (gender ratio: men 55.7% and women 44.3%).

In the present study, females diagnosed with COVID-19 were older than males. For COVID-19 cases in which at least one symptom was observed, the relationship between symptoms was tested based on the phi coefficient (an analogue of Pearson’s correlation coefficient for pairwise comparison of binary variables; Figure 2). The results of the data analysis showed that the appearance of symptoms is not related to each other. The presence of any symptom had little effect on the appearance of another clinical symptom since the phi value does not go beyond the interval from -0.2 to 0.2. A weak positive relationship was detected between fever and other symptoms, such as muscle pain (0.1), cough (0.04), and shortness of breath (0.08). Sore throat (-0.12) and runny nose (-0.11) showed a weak negative association with the fever. Regarding muscle pain, there was a weak positive relationship between shortness of breath (0.08) and diarrhoea (0.03). At the same time, muscle pain had a weak negative association with a runny nose (-0.01). As for the cough, this symptom had only a weak positive association with shortness of breath (0.09), and a weak negative relationship with a sore throat (-0.16), runny nose (-0.08), and diarrhoea (-0.01).

 A weak negative relationship was observed between shortness of breath and such symptoms as sore throat (-0.13), runny nose (-0.01), and diarrhoea (-0.02). According to the results, sore throat had a weak negative association with a runny nose (-0.02), as well as diarrhoea (-0.02). However, the symptom of runny nose had only a weak positive relationship with diarrhoea (0.04).

Out of 5,119 cases of COVID-19, 49 deaths were recorded; therefore, the case fatality rate was 1 %. A two-dimensional analysis was conducted to find the relationship between the prevalence of deaths and demographic characteristics and the presence of concomitant diseases. Statistically significant relationships were found out between the rate of mortality with age, the presence of cardiovascular diseases, hypertension, diabetes mellitus, obesity, kidney, liver, and respiratory diseases (Table 3). It should be noted that the likelihood of most comorbidities also depends on age, and some patients may have several comorbidities. Therefore, the data were analyzed using the logistic regression method, which simultaneously took into account a set of factors that could affect the case fatality rate. In the logistic regression model, the factors having a statistically significant relationship with death included gender (the probability of death among males is 2.6 times higher compared to females), the presence of cardiovascular diseases (the probability is higher by 4.0 times), diabetes (2.4 times, borderline statistical significance), kidney diseases (5.9 times) and respiratory diseases (2.6 times), age (each additional year increased the probability by 1.06 times). Age in the logistic regression model was included as a quantitative variable. The respective OR 1.06 means that a person aged X+1 years has a 1.06 times higher probability of death compared to a person aged X years.

**Table 2:** Presence of COVID-19 symptoms at the time of registration in terms of gender and age

**Symptoms**	**n**	**Any symptom**	**Fever**	**Sore throat**	**Rhinorrhoea**	**Cough**	**Muscle pain**	**Diarrhoea**	**Dyspnoea**
**Age (year)**									
<10	369	87	29	33	17	46	4	9	3
10-19	419	120	29	64	31	59	4	5	7
20-29	1496	496	125	259	151	244	32	26	27
30-39	1016	388	122	205	77	219	40	29	28
40-49	725	282	100	131	41	178	34	14	29
50-59	644	269	107	107	33	180	20	18	36
60-69	242	110	44	41	7	74	10	5	34
Over 70	178	95	29	30	9	60	17	4	38
γ-coefficient		0.18	0.23	0.06	0.13	0.24	0.30	0.09	0.53
P-value		0.001	0.001	0.010	0.001	0.001	0.001	0.100	0.001
**Gender**									
Male	2849	945	309	446	197	497	68	47	101
Female	2267	910	280	427	172	566	93	63	103
Phi		-0.07	-0.02	-0.04	-0.01	-0.09	-0.05	-0.04	-0.03
P-value		0.001	0.095	0.003	0.100	0.001	0.001	0.005	0.070
Total	5116	1855	589	873	369	1063	161	110	204

**Table 3:** The relationship between death and risk factors among patients with COVID-19

**Characteristics**	**Died**	**Alive**	**OR (95% CI)**	**P-value**	**AOR (95% CI)** ^a^	**P-value**
Age (year)	65.2	34.6	1.09 (1.07, 1.11)	0.001	1.06 (1.04, 1.09)	0.001
Gender						
Female	19	2248	1.00		1.00	
Male	30	2819	1.26 (0.71, 2.24)	0.433	2.57 (1.28, 5.17)	0.008
Residence						
Rural	10	1027	1.00		1.00	
Urban	39	4005	1.00 (0.50, 2.01)	1.000	0.71 (0.32, 1.57)	0.396
Cardiovascular disease						
No	14	4769	1.00		1.00	
Yes	32	253	43.1 (22.7, 81.8)	0.001	4.04 (1.42, 11.53)	0.009
Hypertension						
No	15	4694	1.00		1.00	
Yes	31	349	27.8 (14.9, 52.0)	0.001	0.99 (0.35, 2.80)	0.982
Diabetes						
No	30	4930	1.00		1.00	
Yes	17	111	27.8 (14.9, 52.0)	0.001	2.36 (1.02, 5.48)	0.046
Obesity						
No	37	4930	1.00		1.00	
Yes	12	72	22.2 (11.1, 44.3)	0.001	2.27 (0.84, 6.15)	0.108
Kidney disease						
No	28	4925	1.00		1.00	
Yes	17	83	36.0 (19.0, 68.4)	0.001	5.86 (2.47, 13.89)	0.001
Liver disease						
No	39	4956	1.00		1.00	
Yes	6	55	13.90 (5.60, 34.1)	0.001	2.70 (0.71, 10.23)	0.144
Respiratory disease						
No	28	4706	1.00		1.00	
Yes	16	214	12.6 (6.70, 23.6)	0.001	2.56 (1.11, 5.92)	0.028

^a^ Adjusted odds ratio

 Factors such as the presence of hypertension and liver disease, as well as obesity, were found to have a positive relationship with death; however, it was not statistically significant (P<0.1).

## Discussion

 This study analyzed the epidemiological and clinical characteristics of COVID-19 infection among patients of all ages in the territory of the Republic of Kazakhstan. The results demonstrated a high proportion of patients with asymptomatic (40.9%) and mild (36.7%) types of COVID-19. The proportion of temporarily asymptomatic patients (who developed the symptoms later) was estimated at 20% in the current study.

It has been established that the duration of the incubation period was about 14 days (5 days on average) ^32^. Therefore, there was a high risk of coronavirus transmission from person to person, even among asymptomatic virus carriers ^33-36^.

Amongst all clinical features, cough and fever were the most prevalent symptoms, in contrast to relatively rare symptoms, such as diarrhoea, muscle pain, etc. ^37,38^. It should be noted that cough, sore throat, fever, and runny nose were the most prevalent symptoms in COVID-19 patients in the Kazakhstan population. According to recent publications, some syndromic symptoms, such as dry cough, shortness of breath, muscle pain, fatigue, and anorexia are more definite symptoms for the diagnosis of COVID-19 patients than the presence of sore throat, runny nose, vomiting, nausea, diarrhoea, and loss of consciousness ^39^.

Based on the analysis of the age and gender pyramid, a high proportion of those infected with COVID-19 were in the age range of 20-29 years. In a survey study conducted in the United States, respondents aged 18-29 had a higher likelihood of close contact with potentially infected people, compared to those over 50 years ^40^. In addition, it was revealed that respondents who had complaints of coughing more often had close contact with people outside the family environment. It can be explained by the fact that young people were predisposed to COVID-19 infection due to non-compliance with social distancing and other preventive measures.

According to the obtained results in the present study, coronavirus infection was 11.4 % less prevalent in females, and the rate of coronavirus infection in females amounted to 44.3%. Such a situation can be explained by a few factors, including a more adaptive immune response to viral infections in females. Moreover, other factors, such as the effects of hormones (oestrogen) play a crucial role in the resistance to COVID-19^41^. There are a plethora of reports on higher COVID-19 mortality rates among males compared to females^42^. The results of these studies indicate that the male gender acts as a risk factor and increases the possibility of death from coronavirus infection by 2.6 times, compared to the female gender.

In the context of the complications, pneumonia was diagnosed in almost 1 out of 5 of cases (18.9 %), ARDS syndrome was recorded in 1.2 % of patients, and mechanical ventilation was required in 1.3 % of all COVID-19 cases. The study results suggested that the risk of death from COVID-19 increased with age ^43^.

Apart from the age, it was previously reported that the development of COVID-19 severe forms is associated with concomitant conditions, such as hypertension, diabetes, cardiovascular, and cerebrovascular diseases ^44-46^. A relationship was observed between the age and the prevalence of symptoms in the present study. This association was detected in patients in the age group over 75 years for whom the presence of clinical symptoms of COVID-19 was recorded in 53.4% of all the cases.

Other than the association between symptoms and age, it was found that other symptoms, such as shortness of breath, muscle pain, cough, and fever were most prevalent in older patients as well. This result was in line with those of other studies that indicated symptoms such as shortness of breath (76%), fever (52%), and cough (48%) were most prevalent in the group of older patients. Moreover, 33% of the elderly were diagnosed with chronic obstructive pulmonary disease and diabetes, while 43% and 48% of all patients had congestive heart failure and chronic kidney disease, respectively ^47^. These findings once again proved that the severity of symptoms had a positive association not only with the patient’s age but also with the presence of concomitant diseases ^48,49^.

Based on the results of another study, factors, such as the age over 65 years and the presence of cardiovascular diseases increase the frequency of deaths caused by COVID-19 several times ^50^. It conforms to the findings of the present study in which a high mortality rate and a high level of fatal outcomes (11.2%) were observed in patients over 60 years old and those with cardiovascular pathology, respectively. The overall mortality rate from COVID-19 was 10.5% ^51^ in the presence of cardiovascular disease in a recent large study of COVID-19 infection conducted in China.

As indicated by the results of the present study, the presence of chronic kidney increased the risk of the lethal outcome by 5.9 times. According to the results of a previously published study, chronic kidney disease triples the risk of developing severe forms of coronavirus infection and increases the need for treatment in the intensive care unit by 12 times compared to patients without renal pathology ^52^. It should also be noted that there were cases of patients with several concomitant diseases which potentiated the development of complications.

In this study, the fatality rate from COVID-19 was 1% (n=49) versus 99% of those who recovered. Despite the relatively low level of registered mortality from coronavirus infection, it is necessary to take into account the vulnerability of the elderly population and the long-term effects on their health. In fact, the results of recent studies showed a greater risk of death and a low survival rate among the elderly, males, and those with comorbidities ^53^.

Moreover, COVID-19 remains a healthcare burden and a threat to public health due to the lack of reliable and available preventive agents. Another huge problem is the constant mutation of COVID-19 and the development of new strains that can be even more contagious ^54-57^.

 Regarding the limitations of the present study, it should be mentioned that the results of some laboratory studies and treatment regimens were not covered due to the retrospective nature of the presented analysis of verified coronavirus infection cases in Kazakhstan.

## Conclusions

 The analysis of confirmed cases of coronavirus infection among the Kazakhstan population revealed a high proportion (40%) of the asymptomatic cases. The severity of COVID-19 symptoms was directly related to the increase in the age of patients and the presence of comorbidities that can increase the risk of critical outcomes. The case fatality rate due to coronavirus infection was 1% in the infected population. Therefore, the group of elderly patients should be constantly monitored, especially those with concomitant diseases.

## Acknowledgments

 The authors are grateful to the officials in S. D. Asfendiyarov Kazakh National Medical University for their administrative and technical support.

## Conflict of interests

 The authors declare that they have no conflict of interest regarding the publication of the present study.

## Funding

 The study was supported by the National Program for the Introduction of Personalized and Preventive Medicine in the Republic of Kazakhstan (2021–2023).

## Highlights

- A high proportion (40%) of COVID-19 cases in the Kazakhstan population were asymptomatic.
- The severity of COVID-19 symptoms and lethality were directly related to the age of patients and the presence of comorbidities.
- The case fatality rate of COVID-19 in Kazakhstan in the study period was 1 % (n=49) versus 99 % recovered.
